# Salivary neutrophil sampling feasibility in general population for gene expression analysis

**DOI:** 10.1186/s13104-022-06149-2

**Published:** 2022-07-16

**Authors:** Kenneth Rachlin, Helané Wahbeh, Arnaud Delorme, Dean Radin, Loren Carpenter, Salma Ahmadzai, Serena Valletta, Garret Yount

**Affiliations:** 1 Institute of Noetic Sciences, Petaluma, CA USA; 2 University of California, San Diego, CA USA; 3 Chatham University, Pittsburgh, PA USA

**Keywords:** RNA, Noninvasive, Gene expression, Salivary neutrophils

## Abstract

**Objective:**

Human gene expression studies typically rely on peripheral blood samples as a cellular source, however there are numerous situations in which venipuncture is contraindicated. To this end, an oral rinse-based method for collecting salivary neutrophils as a cellular source for gene expression analyses was previously developed and shown in a pilot study with five male participants to yield mRNA expression results comparable to those obtained from peripheral blood samples. The objective of the current study was to characterize the generalizability of the oral rinse-based method by analyzing unpublished RNA quality data obtained through a parent study that collected salivary neutrophil samples using the method from a larger sample size and including both men and women.

**Results:**

The 260/280 nm absorbance ratios of the RNA obtained from 48 participants using the oral rinse-based method were within the expected range (average = 1.88 ± 0.16) for the majority of the samples, and no significant differences in RNA quality were found between participants’ health, age group, or gender. Together with published data confirming the integrity of RNA obtained using the same method, these results support the feasibility of using this noninvasive method for obtaining samples for human gene expression analyses.

## Introduction

Venipuncture is often an unwelcome part of research examining the regulation of human gene expression for various reasons. One reason is that puncturing the skin with a needle to take blood from a vein can confound results by triggering local cellular transcriptional changes in genes of interest, such as cytokines [1, 2] and hormones [3]. The risk of infection any time the skin is broken and needle phobia [4–6] are reasons to avoid venipuncture from the perspective of the participants. These considerations have prompted the use of neutrophils as a cellular source in human gene expression studies that can be collected without a needle stick [7] For example, Roy and colleagues [8] collected neutrophils noninvasively at epidermal wound sites to assess stimulus-transcription coupling associated with psychological stress however such protocols have limited applicability due the requirement that participants have preexisting wounds.

Our group developed a truly noninvasive, oral rinse-based protocol for collecting salivary neutrophils and demonstrated in a pilot study with five male participants that high-quality RNA can be harvested from the samples [9]. The pilot study used electrophoretic separation of total RNA to verify the integrity of the RNA collected by the noninvasive protocol. Using qRT-PCR analysis, the pilot study also demonstrated that the mRNA harvested from the samples yielded results that were comparable to those obtained from peripheral blood samples collected in parallel through venipuncture from the same participants. Messenger RNA levels for IL-8, IL1b, NAMPT, and beta-actin were detectable within the range typically found in gene expression analyses (Ct < 30) for both sample types.

The current study assesses the generalizability of the oral rinse-based method by analyzing unpublished RNA quality data obtained using the method in a parent study [10] that collected duplicate samples from 48 men and women. The parent study used a direct detection, multiplex analysis system (nCounter^®^, Nanostring, Washington, WA) to quantify mRNA expression levels for a panel of neuroinflammatory genes. Three house-keeping genes were selected for normalization of target gene expression levels based on their consistent expression levels across all of the mRNA samples: CNOT10, GUSB, and TADA2B. The current study focuses on testing whether RNA quality differed among demographic groups participating in the parent study.

## Main text

### Methods

The parent study recruited adults experiencing chronic hand and wrist pain and assessed a range of outcome measures before and after an experimental intervention. A total of 48 participants provided oral rinse samples on two occasions, separated by 3 weeks (total of 96 samples). Participants were asked to refrain from eating or drinking anything other than water for two hours prior to sample collections in a treatment room. At the time of collection, participants rinsed for 30 s and then expectorated two 15 ml aliquots of HBSS plus 2 mM calcium and 0.4 mM magnesium at pH 7.4 into a 50 ml tube. The tubes were kept on ice and transferred to the laboratory for processing within 10 min.

The oral rinse procedure initially yielded a mixture of exfoliated cheek epithelial cells and salivary neutrophils that were subsequently separated by filtration according to their consistent size difference (cheek epithelial cells average 50–60 μm in diameter and neutrophils average 12–15 μm). The separation procedure was adapted from a published dental protocol [11] and involved sequential passive filtration through a 20 μm and a 10 μm nylon mesh. The final filtrate that was centrifuged at 800 rcf, 4 °C, for 7 min to pellet the purified neutrophils. The supernatant was aspirated and the cell pellet was resuspended in 1 mL of the HBSS solution. A 10 μL aliquot was removed for cell counting using a hemocytometer and the remaining sample was transferred to a 1.5 mL microcentrifuge tube and centrifuged at 800 rcf, 4 °C, for 10 min. The final supernatant was aspirated and the cell pellet (92–98% pure neutrophils) were resuspended in RNAlater (Invitrogen, Waltham, MA) to preserve the integrity of the RNA and stored at 4 °C until transferring to a service laboratory for spectrophotometric analysis (Core Diagnostics, Hayward, CA). The primary outcome considered in the analysis of the samples was the ultraviolet 260/280 nm absorbance ratios for all 96 samples. Additional analysis included testing for differences in RNA quality between demographic groups, including self-reported health, age group, and gender.

## Results and discussion

**Fig. 1:**
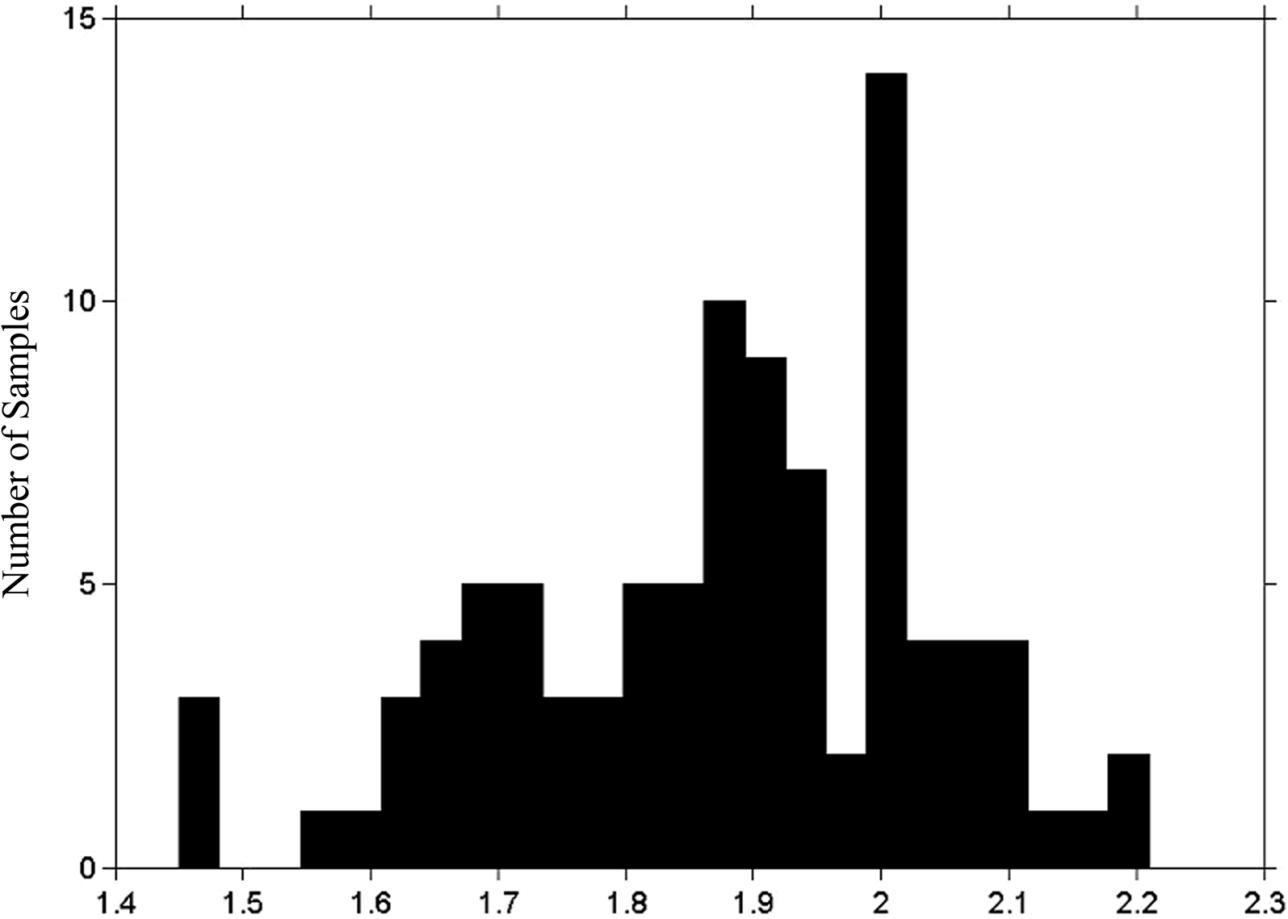
RNA Ultraviolet 260/280 nm Absorbance Ratios for all Samples

All of the participants were able to follow the collection procedures easily without any discomfort or distress. The average number of salivary neutrophils collected was 2.3 × 10^6^ and the average yield of total RNA was 431 ng, which are sufficient quantities for most gene expression analysis technologies. Figure 1 depicts the ultraviolet 260/280 nm absorbance ratios for all 96 samples. Historically, this ratio has been used as an indicator of purity for RNA and a ratio of ~ 2.0 is generally considered “pure” but accepted values typically range from (1.8–2.1). Most of the samples had absorbance ratios within this range but ratios for 22 samples fell below the 1.8 threshold. Depending upon the downstream analysis technology, these samples might require a second round of RNA purification.

To test for differences in RNA quality (based on absorbance spectra) between demographic groups, a one-way ANOVA was performed with multiple comparisons for reported health, age group, and gender. The number of samples in each group, the means, and standard error are reported as well as the F-statistics (See Table 1). No significant differences in RNA quality were found between demographic groups, thus these data provide further support for the feasibility of using the oral rinse-based method for obtaining samples for human gene expression analyses in the general population.

**Table 1:** RNA Ultraviolet 260/280 nm Absorbance Ratios by Demographic Category

				ANOVA
Health	n	A 260/280 mean	SE	*F*(4,91) = 1.27, *p* > .05
N/A	24	1.88	0.033	
Fair	16	1.82	0.041	
Good	22	1.92	0.035	
Very Good	28	1.89	0.031	
Excellent	6	1.80	0.066	
Age				*F*(5,90) = 1.41, *p* > .05
20–30	4	1.72	0.081	
30–40	14	1.91	0.043	
40–50	8	1.92	0.057	
50–60	14	1.91	0.043	
60–70	34	1.88	0.028	
70–80	22	1.84	0.034	
Gender				*F*(1,94) = 2.16, *p* > .05
M	16	1.82	0.041	
F	80	1.89	0.018	

## Limitations

- The sample size was relatively small.
- The current study did not compare expression levels obtained using the oral rinse-based method with isolation from peripheral blood sample. However, our previous report [9] did so by confirming that the mRNA expression levels for four target genes measured from neutrophil samples collected with the oral rinse-based method were within the same range as those obtained from peripheral blood samples collected from the same individuals at the same time.
- Regarding the application of the oral rinse-based method, the serial filtration procedure does not exclude microorganisms and viruses present in the saliva. Depending upon the RNA targets being investigated, these contaminants may require removal to eliminate confounding signals. One method to achieve such removal is to include an additional filtration step to separate the neutrophils away from microorganisms and viruses according to their smaller sizes (both < 1 μm). Unlike the filtration process used in this study to isolate neutrophils away from cheek epithelial cells, this additional filtration step (e.g., with a 1 μm nylon mesh) would retain the cells of interest (neutrophils) and the filtrate would be discarded.

## Data Availability

All data generated or analyzed during this study are included in this published article and available online at Open Science Framework: https://doi.org/10.17605/OSF.IO/JTEG9

## Abbreviations

CNOT10: CCR4-NOT transcription complex, Subunit 10
Ct: Cycle threshold
GUSB: Glucuronidase beta
HBSS: Hank’s balanced salt solution
IL1b: Interleukin-1 Beta
IL-8: Interleukin-8
mRNA: Messenger RNA
NAMPT: Nicotinamide phosphoribosyltransferase
qRT-PCR: Quantitative reverse-transcription polymerase chain reaction
RNA: Ribonucleic acid
TADA2B: Transcriptional adaptor 2B
UV: Ultraviolet
